# Efficacy of the School Gambling Prevention Program “What’s at stake?”: A Cluster Randomized Controlled Trial

**DOI:** 10.1007/s10899-026-10486-3

**Published:** 2026-04-18

**Authors:** Daniel Lloret-Irles, Víctor Cabrera-Perona

**Affiliations:** Department of Health Psychology, Miguel Hernández University, Elche, Spain

**Keywords:** Gambling, Adolescent, School prevention, Efficacy, Assessment

## Abstract

Adolescent gambling has become a growing public health concern. Despite international evidence supporting preventive interventions, few programs have been rigorously evaluated in Spanish-speaking populations. This study examined the effectiveness of the school-based gambling prevention program *What’s at stake?* (*WAS?*). A randomized controlled trial was conducted with secondary school students (*N* = 6,308; 50.6% female; mean age = 15.3 years). Participants were assigned to an experimental group (*n* = 5,022) or control group (*n* = 1,286). Assessments were carried out at baseline (T_1_) and one week post-intervention (T_2_). Primary outcomes included gambling intention and gambling severity. Secondary outcomes included gambling motives, cognitive bias, normative perception, and risk perception. Validated self-report measures were employed. Population-averaged pre-post effects were estimated using generalized estimating equations. The pre-post analyses showed significant population-average differential changes with small standardized magnitudes (RESI) in motivational dimensions, normative perception, risk perception, and gambling intention. Regarding intention, subgroup analyses by gender showed similar patterns. Descriptively, the proportion of at-risk and problem gamblers decreased in the experimental group at post-intervention, whereas minimal change was observed in the control group. The *WAS?* program produced significant short-term population-level preventive effects on key cognitive, motivational and intentional risk factors for gambling. Our findings contribute to strengthening the evidence base for culturally and linguistically adapted gambling prevention and extend program benefits in Hispanic adolescent populations. Future studies should include follow-up assessments and booster sessions to evaluate the maintenance of these effects.

Over the past decade, gambling has evolved into a digitally networked and globally normalised activity that poses growing challenges to population health (Wardle et al., 2024). This transformation is not merely technological but structural. Online gambling platforms, mobile applications, sponsorships embedded in sports, the use of celebrities and new forms of online marketing on social media have created a vast commercial ecosystem that normalises gambling and magnifies consumer exposure (Ukhova et al., 2024; Wardle et al., 2019, 2024). These elements normalises gambling behaviour, and substantially magnifies exposure among young people and adults alike (Ukhova et al., 2024; Wardle et al., 2019, 2024).

Thus, a comprehensive meta-analysis estimates that nearly half of adults and one in five adolescents worldwide report gambling in the past year. This percentage increases to 33.7% in North America (Tran et al., 2024). Parallel trends are observed in Europe: the proportion of 15-year-old European adolescents who reported gambling in the past 12 months has increased by 57% in recent years, rising from 14% in 2015 to 22% in 2019 (ESPAD Group, 2016, 2020). These data suggest that the proliferation of online gambling opportunities has been accompanied by a measurable increase in engagement across age groups, particularly among younger populations.

This growing exposure is concerning because gambling constitutes a recognised public health problem. Harms and adverse effects extend far beyond diagnosable gambling disorders and encompass a broad spectrum of negative outcomes, including physical and mental health, financial strain, interpersonal relationships, family conflict, reduced academic performance, work-related problems and social functioning (Abbott, 2020; The Lancet, 2023; Wardle et al., 2019).

Epidemiological evidence confirms that gambling is not only widespread but also increasingly concentrated among vulnerable groups. Among these, adolescents are especially exposed: developmental sensitivity to reward, combined with targeted digital marketing and gambling-like features in gaming environments, has blurred the distinction between play and wagering and makes them particularly susceptible to risky decision-making or normative misperceptions (Di Censo et al., 2024; Neighbors et al., 2015). This convergence of psychosocial vulnerability and technological accessibility underscores the need for early, population-based prevention (Davis et al., 2025; Ukhova et al., 2024). This vulnerability is reflected in prevalence data. Thus, the proportion of European adolescents engaging in problematic gambling behaviour is 7.8% (11% boys and 4.6% girls) (ESPAD Group, 2024). A meta-analysis reviewing studies conducted in 23 countries since 2000 estimated that between 0.2% and 12.3% of European adolescents would meet criteria for problem gambling (Calado et al., 2017), a prevalence substantially higher than that found among adults (Gupta et al., 2013). Early onset of gambling increases the risk of developing addictive behaviours in adulthood (Dussault et al., 2011; El-Guebaly et al., 2015; Williams et al., 2015).

To address the psychological, social, and contextual mechanisms that shape gambling behaviour (Ukhova et al., 2024), it is important to develop preventive interventions specifically designed for adolescents and young people to reduce or avoid gambling during adolescence. There is growing research on the effectiveness of gambling prevention programs. To date, eight systematic reviews have been identified. Ladouceur et al. (2013) published the first systematic review including 15 studies: although interventions reduced cognitive distortions and increased knowledge, the lack of follow-up and behavioural outcomes prevented solid conclusions. Keen et al. (2017) reviewed 19 studies across 15 programs: all reported cognitive improvements, but only nine assessed behavioural changes and five found significant effects. Limitations persisted, such as short follow-ups, lack of control groups, and measurement errors. Oh et al. (2017) analysed 14 programs and highlighted the importance of including audiovisual materials, intensive sessions, and trained facilitators. Forsström et al. (2021), applying stricter criteria, conducted a meta-analysis of six adolescent studies and one in university students: they found preventive effects on behaviour, though with low evidence; cognitive outcomes showed very low evidence. Giménez-Lozano and Morales-Rodríguez (2022) reviewed 15 studies and confirmed short-term cognitive improvements but noted the same shortcomings: lack of follow-ups, focus on cognitive variables, and misclassification errors. St Quinton et al. (2022) analysed techniques used in 16 studies and identified promising ones: providing background information; conducting behavioural experiments; offering information on social and environmental consequences; and informing about emotional consequences. Monreal-Bartolomé et al. (2023) analysed 32 studies of 28 programs. The objectives included raising awareness of gambling consequences, correcting biases, fostering coping skills, critical thinking, and self-control. Activities encompassed debates, presentations, testimonies, and videos. At the cognitive level, misconceptions and fallacies were reduced; affectively, negative attitudes toward gambling were promoted; and behaviourally, 84% of the studies reported reductions in frequency and severity of gambling. Finally, Talebi & Bazrafshan (2025) conducted a meta-analysis of 15 studies from 2002 to 2022, finding a significant reduction in gambling behaviour, with greater effects for social skills training programs. However, heterogeneity was high, and the overall quality of evidence was very low. Taken together, the evidence points to primarily cognitive and affective benefits, with promising but still limited behavioural effects due to methodological shortcomings. These reviews underscore that effective prevention requires a paradigm shift, from treating gambling as an individual behavioural failure to addressing it as a psycho-socially and commercially conditioned phenomenon (Davis et al., 2025). In this sense, theory-driven, context-sensitive and participatory school-based interventions grounded in behavioural models, linking cognitive, motivational, and social dimensions of behaviour change, can provide this integrative approach.

To our knowledge, only three studies have evaluated the effectiveness of gambling prevention programs in the Spanish population (Chóliz et al., 2021; Giménez-Lozano et al., 2022; Lloret & Cabrera-Perona, 2019). Evidence on the effectiveness of school-based gambling prevention programs is almost exclusively Anglophone, with the United States, Australia, Canada, and the United Kingdom accumulating most of the research (Forsström et al., 2021; Monreal-Bartolomé et al., 2023; Talebi & Bazrafshan, 2025). Therefore, due to a lack of research on gambling prevention, mainly in Hispanic culture, conclusions must be read cautiously, considering the challenges of cultural adaptation.

The goal of the present study is to analyse the effectiveness of a gambling-specific prevention program: “What’s at stake?”. Specifically, the study aims to examine population-average differences in change between the experimental and control conditions across pre and post-intervention measures. This program represents a structured, theoretically coherent response—one that aligns psychological capability, opportunity, and motivation as modifiable determinants of gambling intention and behaviour in adolescence. The program targets several misconceptions, the illusion of control, cognitive biases in gambling, attitudes toward gambling advertising, and ultimately gambling behaviour.

## Method

### Study Design and Participants

A cluster randomized controlled trial with an individual matched pre-post panel design was conducted. The unit of randomization was the classroom within each participating school.

Adolescents attending secondary school attending public schools from different regions of Spain were recruited for the study. Participants were students in 3rd, 4th and 1st BACH grade of compulsory secondary education (ESO, BAT) approximately 14–17 years old.

Regarding inclusion criteria classroom was eligible if: a) It belonged to a school that agreed to participate; b) School leadership and the class tutor authorized implementation during regular instructional time. Each student, were included if they: c) provided written informed assent (and parental/guardian consent for minors), d) were able to understand and complete the self-report measures in Spanish without assistance.

Regarding Exclusion criteria were: a) documented cognitive or learning difficulties that prevented comprehension of the intervention or questionnaires, b) absence on the baseline assessment day, c) prior exposure to the full “What’s at stake?” program during the same academic year.

Individual responses were linked from baseline to post-intervention using a deterministic anonymous composite identifier constructed from non-identifiable administrative codes (municipality, school, classroom) combined with an anonymous individual questionnaire identifier.

Data were collected at two time points: before the intervention to establish baseline (T_1_), one week after the intervention (T_2_). Sample sizes at each wave were 5,022 and 4,303 for the experimental group, and 1,286 and 686 for the control group at T_1_, T_2_, respectively. The retention rate between baseline and post-intervention was 86% for the matched pre-post sample. The originally planned six-month follow-up wave was not included in the main analyses due to substantial attrition and insufficient statistical power.

Some questions were only answered if participants reported having gambled, such as gambling severity and the four types of gambling motivation, which resulted in reduced sample sizes for these variables (Table 1). The mean age at T_1_ was 15.33 years (range 14–17, SD = .95), and 50.6% were female. Baseline distributions were comparable across conditions for gambling measures and sociodemographic variables, except for age, where the control group was slightly younger than the experimental group, although the effect size was trivial (Table 1).

**Table 1:** Baseline differences between experimental and control group

	Condition	*N*	Mean	SD	t	df	*p*	d	CI
Age	Experimental	5022	15.390	.941	6.279	6306	< .001	.196	[.13; .24]
	Control	1286	15.210	.959					
Intention	Experimental	5022	2.073	1.327	1.323	6306	.093	-	[-.026; .14]
	Control	1286	2.018	1.321					
Severity	Experimental	699	1.612	1.807	1.586	859	.057	-	[-.059; .56]
	Control	162	1.364	1.733					
Motivation Social	Experimental	1739	.685	.690	-.906	2136	.182	-	[-.11; .042]
	Control	399	.721	.804					
Motivation Excitement	Experimental	1739	.838	.904	-.283	2136	.388	-	[-.12; .09]
	Control	399	.853	1.036					
Motivation Coping	Experimental	1739	.340	.672	−1.788	524.058	.037	-.113	[-.17; .01]
	Control	399	.420	.830					
Motivation Financial	Experimental	1739	1.190	1.164	.508	2136	.306	-	[-.09; .16]
	Control	399	1.157	1.188					
Risk Perception	Experimental	5022	1.183	.732	1.062	1872.968	.144	-	[-.02; .08]
	Control	1286	1.157	.800					
Normative Perception	Experimental	5022	1.987	.756	3.343	6306	< .001	.104	[.03; .12]
	Control	1286	1.908	.724					

### Measures

The following self-report measures were administered to participants at baseline (T_1_) and post-intervention (T_2_):

#### Primary Outcomes

*Intention to gamble* was assessed with three items regarding plans to gamble in the near future: “Do you intend to gamble?”, “Do you plan to gamble online soon?”, and “If you had the opportunity, would you like to enter a gambling venue?”. Items were rated on a 7-point Likert scale ranging from 1 (Definitely not) to 7 (Definitely yes). Internal consistency was Cronbach’s α = .79.

*Gambling severity* was assessed using the South Oaks Gambling Scale–Revised for Adolescents (SOGS-RA) (Winters et al., 1993), Spanish version by Becoña (1997). The SOGS-RA assesses gambling behaviour and related problems over the previous 12 months. This instrument includes 12 dichotomous (Yes/No) items, adapted from the SOGS (Lesieur & Blume, 1987). Reliability in the study sample was Cronbach’s α = .83. The SOGS-RA yields three categories according to the cut-off scores suggested by Winters et al. (1993): 0–1 = non-gambler or no gambling problems; 2–3 = at-risk gambler; and ≥ 4 = problem gambler.

*Gambling motives*. Gambling motives were assessed using the Gambling Motives Questionnaire – GMQ (Stewart & Zack, 2008; Spanish adaptation by Grande-Gosende et al., 2019), which comprises three dimensions: (1) *Enhancement*, where gambling is motivated by positive internal reinforcement and pleasant emotions; (2) *Coping*, where gambling is conceived as a way to escape; and (3) *Social*, where gambling is perceived as a social activity to spend time with friends. The extended version, GMQ-F (Dechant, 2013), adds a fourth dimension (4) *Financial* motives (gambling for the purpose of winning money). Internal consistency of the total scale and each subscale in this study was Cronbach’s α > .933.

*Risk perception* was measured with the PR subscale of the Early Detection Gambling Addiction Risk Questionnaire – Adolescents (EDGAR-A) (Cabrera-Perona et al., 2022). It consists of 8 items evaluating the belief that gambling entails negative consequences. Scoring is inverted relative to the construct, such that higher scores indicate lower risk perception, and therefore a higher risk of gambling. Responses ranged from 0 (Strongly disagree) to 4 (Strongly agree). Internal consistency was Cronbach’s α = .86.

*Normative perception*. The PN subscale of EDGAR-A (Cabrera-Perona et al., 2022) was used to assess beliefs about the perceived frequency of gambling among peers of similar age. It comprises 4 items, with internal consistency of Cronbach’s α = .71.

### Procedure

This study was approved by the Ethics Committee of the authors’ home institution. Schools were contacted through an institutional invitation. When they agreed to participate, school leadership and parents were informed through a letter sent by each school, emphasizing the confidentiality of the data. Two-stage probabilistic sampling procedure was used. First, secondary schools in the several regions (i.e. Alicante, Valencia, Huelva, Madrid) were randomly selected based on the municipality–school ratio. Secondary schools were chosen as the implementation setting due to the high prevalence and normalization of gambling among adolescents (ages 14–17). Second, within each participating school, classrooms were randomly allocated to either the experimental group (EG) or a wait-list control group (CG). The allocation ratio was approximately 4:1 (EG:CG), reflecting logistical requirements linked to the large-scale educational rollout. This choice was driven by feasibility and ethical constraints from the schools and education authorities, who asked us to maximize the number of classes receiving the intervention. This approach maximized program coverage while preserving a sufficiently sized control group for inferential comparison—an established practice in school-based prevention trials operating under operational constraints.

All participants completed the self-report questionnaires in collective sessions: prior to the first session (T_1_), one week after the last session (T_2_). Participants were informed that their participation was voluntary, that they could withdraw at any time, and that their responses would remain confidential. Intervention sessions were delivered by trained graduate-level facilitators in psychology and education, who completed a standardized 6-h training. Facilitators followed a structured manual and received weekly supervision. Fidelity to the instructional materials was monitored informally; however, session-level fidelity logs were not collected.

#### Intervention and Logical Model

*What’s at stake?* (WAS?) consists of four 50-min sessions implemented in small groups. Sessions were delivered by trained implementers, mainly psychologists specialized in addictive behaviours. The program provides a manual and supporting materials for each session, including videos, presentations, graphics, data, and worksheets. A complete assessment questionnaire is also available. Both the manual and supplementary materials can be freely downloaded from www.programaquetejuegas.org.

*WAS?* aims to achieve five objectives: I. Increase risk perception; II. Reduce motivational beliefs and positive attitudes toward gambling and betting; III. Reduce the illusion of control over gambling success; IV. Identify the manipulative tactics of gambling advertising messages and V. Strengthen self-efficacy to resist peer and social pressure.

The program is grounded in the Theory of Planned Behavior (TPB, Ajzen, 1991) which identify intention as the main predictor of gambling behaviour, itself explained by three key factors: attitudes toward gambling, subjective norms, and self-efficacy, mediating the influence of attitudes, subjective norms, and perceived behavioural control. Similarly, in COM-B model (Michie et al., 2014; West & Michie, 2020), changes in capability, opportunity, and motivation are expected to modify intention before producing behavioural shifts. Consistent with this theoretical framework, gambling intention was designated as the *primary proximal outcome*. Preventive interventions targeting adolescents typically operate on these proximal cognitive mechanisms rather than on gambling behaviour itself, which is often infrequent at this developmental stage. In addition, gambling severity (SOGS-RA) was included as a *primary behavioural outcome* assessed at baseline and post-intervention as it represents the clinically relevant manifestation of harm and aligns with public-health priorities emphasised in recent literature (e.g., Tran et al., 2024; Wardle et al., 2024). Although behavioural changes are not expected immediately after a brief universal school-based program, measuring gambling severity allows for detecting delayed or consolidation effects.

**Fig. 1:**
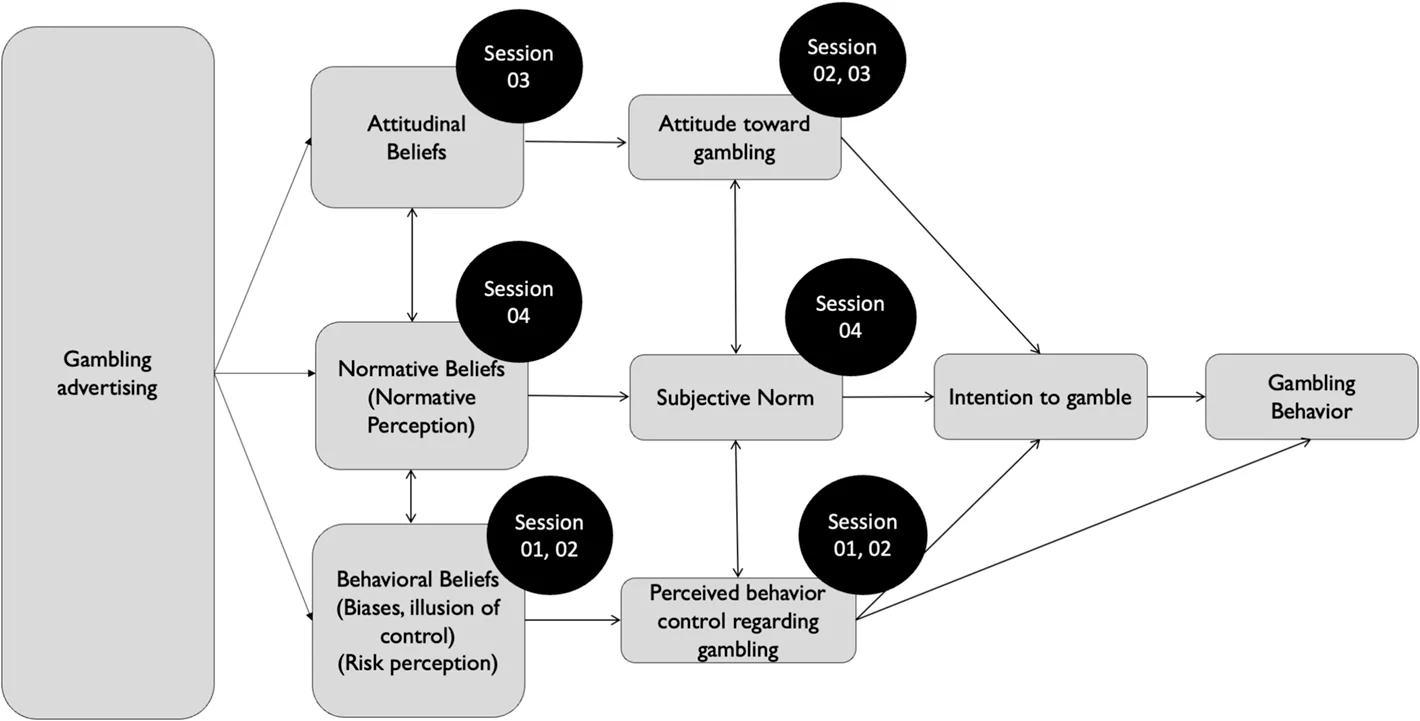
Logic model of the “What’s at stake?” (WAS?) program based on the Theory of Planned Behavior (TPB, Ajzen, 1991)

**Fig. 2:**
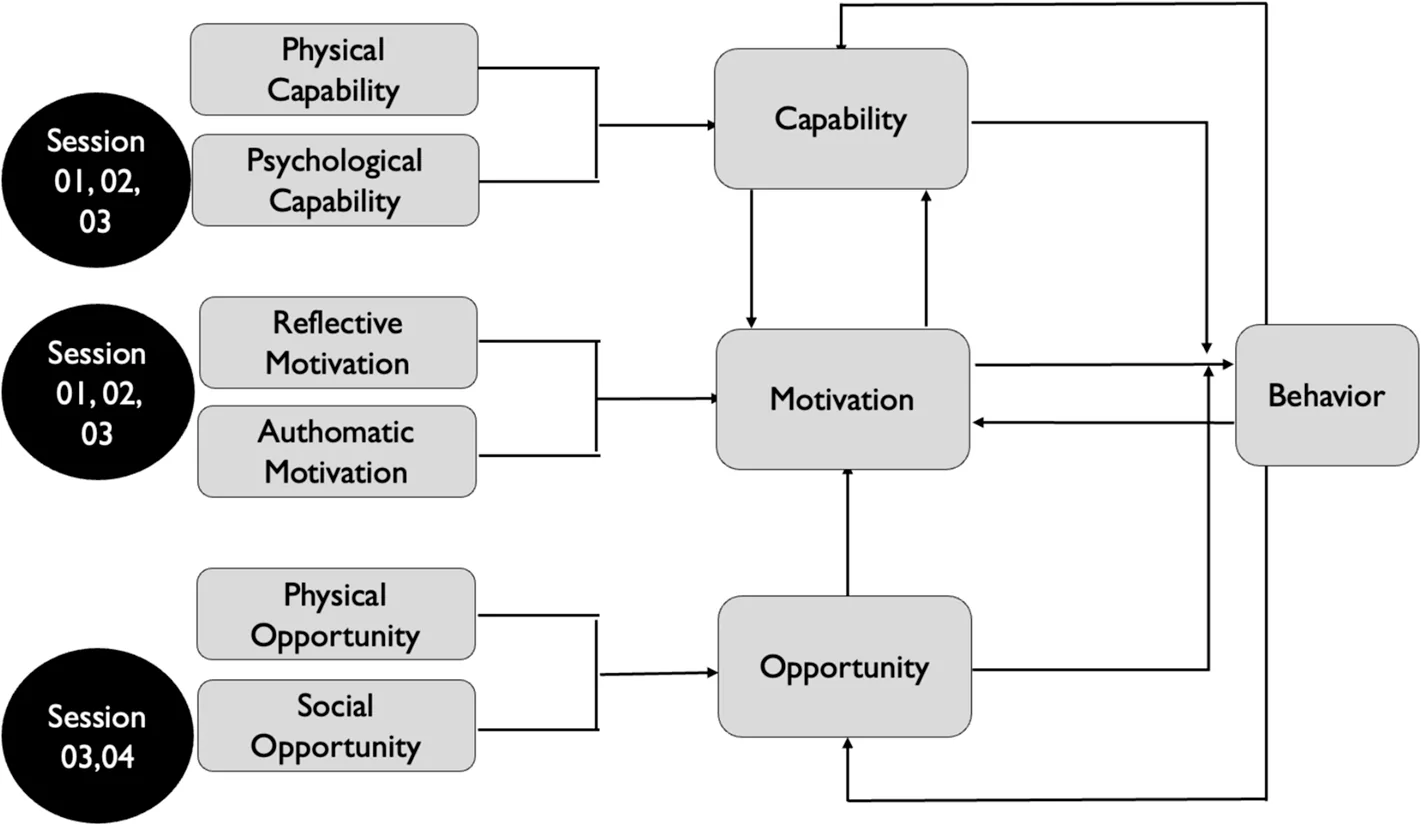
Logic model of the “What’s at stake?” (WAS?) program based on the COM-B framework (Michie et al., 2014; West & Michie, 2020)

Figures 1 and 2 present the graphical logic model of the intervention. Regarding the TPB, Session 1 (increasing risk perception and gambling motivations) targets *behavioral beliefs* related to risk, prompting a reevaluation of gambling consequences and, consequently, a more accurate sense of behavioral control. Session 2 (probability and illusion of control) directly addresses *distorted behavioral beliefs* reinforced by the gambling industry—such as betting systems, cognitive biases, and the illusion of control—thereby promoting a more realistic perception of control. Session 3 (critical reflection on advertising) focuses on *attitudinal beliefs*, challenging the normalization of gambling and the positive—affective and cognitive—attitudes encouraged by advertising and marketing strategies. Finally, Session 4 (normative perception and resistance to peer pressure) targets *normative beliefs* by correcting the perception that gambling is a common or expected behavior among peers. The program also integrates the COM-B model as a comprehensive framework linking behavioral determinants to intervention mechanisms. The four sessions operationalize COM-B components in a complementary way: Session 1 enhances *psychological capability* and reduces *automatic motivation* by increasing risk perception and questioning initial gambling motivations. Sessions 2 and 3 strengthen *reflective motivation* and psychological capability through activities that correct cognitive biases, improve probability understanding, and promote critical analysis of gambling advertising and normalization. Session 4 acts on *social opportunity*, challenging perceived norms and training resistance to peer pressure, thereby increasing self-efficacy and assertive skills. Taken together, these mechanisms aim to reduce gambling intention, the primary outcome according to both TPB and COM-B, and, consequently, to decrease gambling behavior and severity.

What’s at stake? follows four basic principles: non-directive, participatory, reflective, and fully manualized.

### Data Analysis

Pre–post intervention effects were analysed using population-averaged Generalized Estimating Equations (GEE) to account for the dependency of repeated measurements within students. This approach provides valid population-average estimates for intervention effects in cluster-randomized prevention trials with matched observations. Continuous outcomes were modelled assuming a Gaussian distribution with an identity link function. An exchangeable working correlation structure was specified to model within-student dependency between baseline (T_1_) and post-intervention (T_2_), with student identification number specified as the subject variable (id = student). Robust (sandwich) standard errors were used to obtain valid population-average estimates in the presence of within-subject correlation. Time was treated as a categorical factor (baseline vs. post-intervention), and intervention effects were evaluated through the Condition × Time interaction, which represents the differential change between the experimental and control groups from T_1_ to T_2_. All available pre–post observations were included in the analyses.

Model parameters are reported as regression coefficients (β), robust standard errors, 95% confidence intervals, Wald χ^2^ tests, and associated p-values. Effect size magnitude was described using the Robust Effect Size Index (RESI; Vandekar et al., 2020), derived from the GEE models and interpreted analogously to a correlation or Cohen’s d (.10 small, .30–.40 medium, .50 large).

## Results

The pre–post differences between the experimental and control groups are presented according to primary and secondary outcomes.

### Primary Outcomes

#### Intention to Gamble

**Fig. 3:**
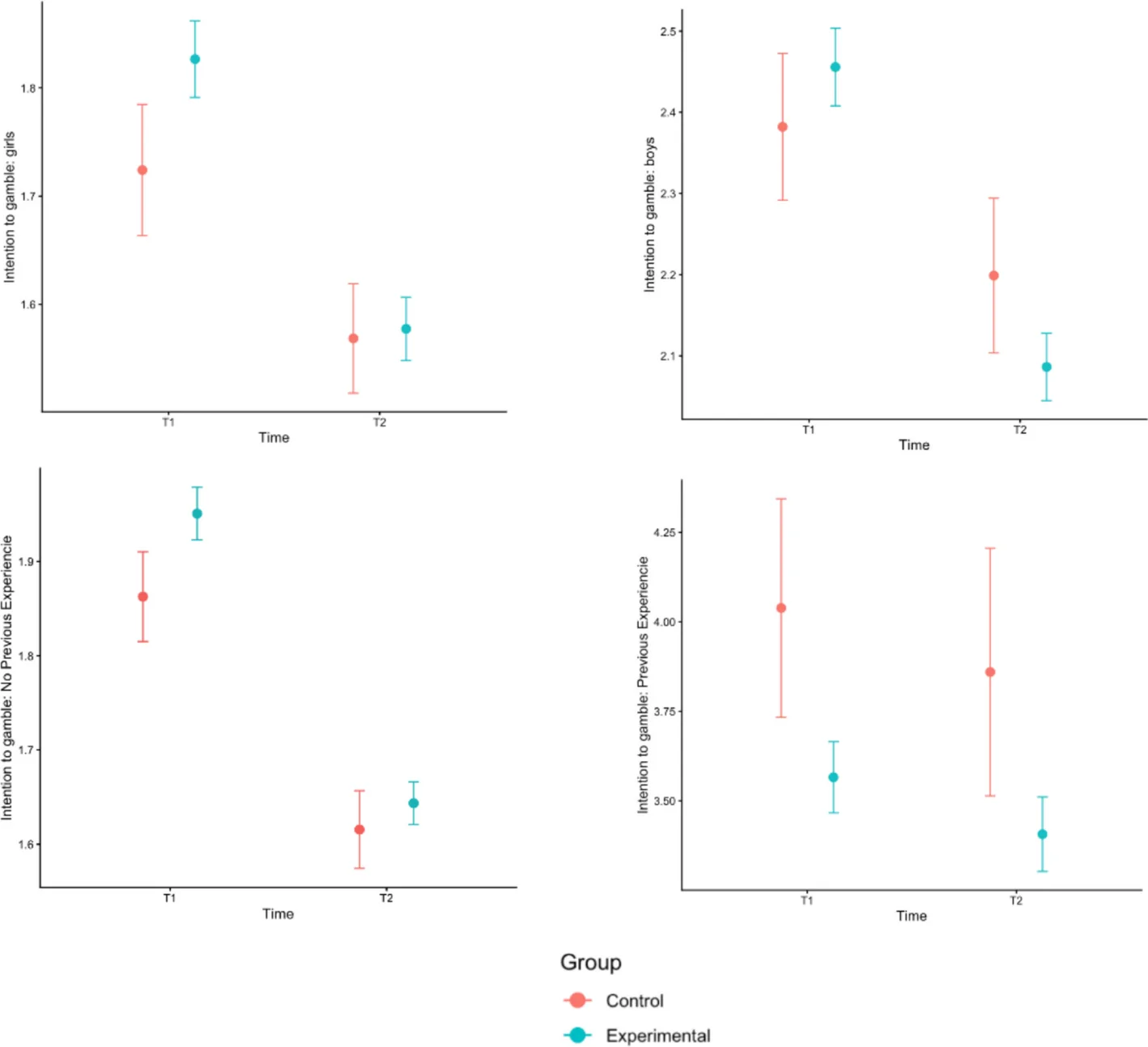
Intention to gamble in experimental and control group

A significant Condition × Time interaction was observed for gambling intention (β = − .12, *p* = .004), indicating a greater reduction in the experimental group than in the control group (Fig. 3). Subgroup analyses by gender showed a similar descriptive pattern.

Subsequently, a comparative analysis was conducted according to prior gambling experience. The Condition × Time interaction was not statistically significant in either the experienced group (β = .02, *p* = .931) or the non-experienced group (β = − .04, *p* = .249), indicating no differential change between the experimental and control conditions within these subgroups (Table 2).

**Table 2:** Intention to gamble. *p*-values associated to each adjusted model GEE

Parameter	Boys	Girls	Prior experience	No prior experience
(Intercept)	< .001	< .001	< .001	< .001
Experimental	.158	.004	.004	.002
T2	.002	< .001	.372	< .001
Experimental*T2	.029	.114	.931	.249

#### Severity of Gambling Behaviour

Changes in gambling severity are presented descriptively based on the SOGS-RA cut-off categories (Table 3). At baseline (T_1_), the distribution of severity levels were similar in the experimental and control groups. 34.8% of the experimental group were classified as at-risk or problem gamblers. Following the intervention, this proportion decreased to 12.8%, while the percentage of participants in the no gambling/no problem category increased from 65.2% to 87.2%. In contrast, the control group showed minimal descriptive change between T_1_ and T_2_.

**Table 3:** Evolution of gambling severity in experimental and control groups

		Experimental			Control	
	No gambling /No risk	Risk	Problematic	No gambling/No risk	Risk	Problematic
T_1_	456 (65.2%)	176 (25.2%)	67 (9.6%)	111 (68.5%)	35 (21.6%)	16 (9.9%)
T_2_	1355 (87.2%)	130 (8.4%)	69 (4.4%)	53 (68.8%)	16 (20.8%)	8 (10.8%)
Boys						
T_1_	282(63.7%)	121(27.3%)	40(9%)	62(68.9%)	20(22.2%)	8(8.9%)
T_2_	615(82%)	91(12.1%)	44(5.9%)	27(67.5%)	7(17.5%)	6(15%)
Girls						
T_1_	169(68.7%)	54(22%)	23(9.3%)	48(69.6%)	14(20.3%)	7(10.1%)
T_2_	696(93%)	37(4.9%)	15(2%)	26(72.2%)	8(22.2%)	2(5.6%)

Descriptive analyses by gender indicated a shift toward lower risk categories in the experimental group at post-intervention. Among boys, the proportion in the no-risk category increased from 63.7% to 82%, accompanied by a decrease in problem gambling (9% to 5.9%). Among girls, a more pronounced descriptive shift was observed, with the no gambling/no-risk category increasing from 68.7% to 93% and problem gambling decreasing from 9.23% to 2% (Table 4).

### Secondary Outcomes

#### Motivation to Gamble

**Fig. 4:**
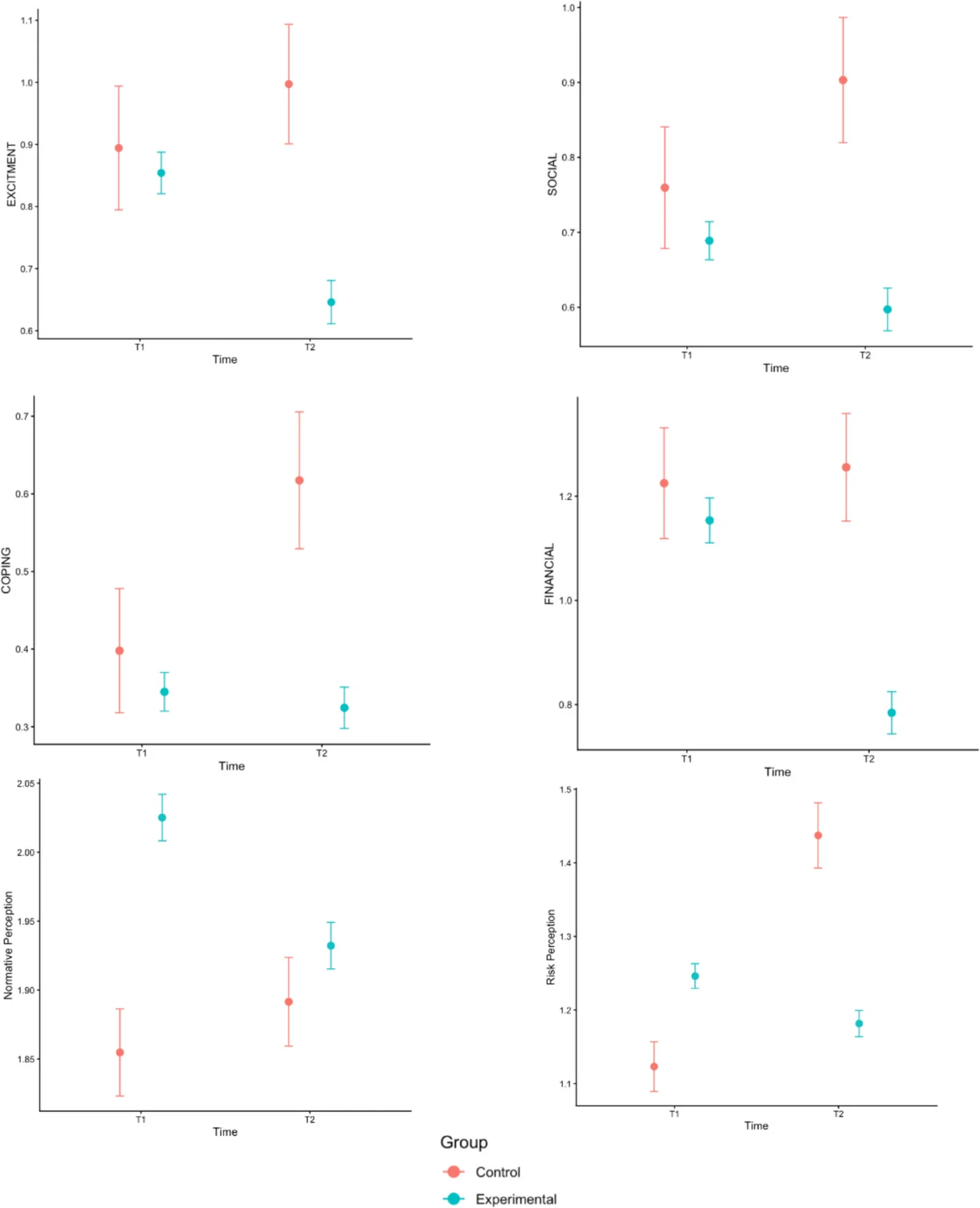
Motivations to gamble and normative and risk perception in experimental and control group

A significant Condition × Time interaction was observed, indicating that motivational scores increased in the control group but decreased in the experimental group from T_1_ to T_2_, although effect magnitudes (RESI) were small (Fig. 4).

#### Risk Perception

A significant Condition x Time interaction was observed (β = − 0.38, *p* < .001) (Table 4). Although the experimental group began at T_1_ with a slightly higher perception of risk than the control group (β = 0.12, *p* < .001), their changes diverged markedly by T_2_. While the control group experienced a notable increase in reverse-scored indicator (β = 0.32), the experimental group showed a slight decrease, which translates into an increase in risk perception.

**Table 4:** Motivation to gamble, risk and normative perception. *p*-values associated to each adjusted model GEE

Parameter	Social	Excitement	Coping	Financial	Risk perception	Normative perception
(Intercept)	< .001	< .001	< .001	< .001	< .001	< .001
Experimental	.142	.592	.273	.39	< .001	< .001
T2	.033	.297	.001	.762	< .001	.209
Experimental*T2	< .001	< .001	< .001	< .001	< .001	< .001

#### Normative Perception

A significant Condition x Time interaction was found (β = − 0.12, *p* < .001) (Table 4), indicating a reduction in normative perception scores in the experimental group, whereas the control group remained stable (or showed a slight non-significant increase) at T_2_.

## Discussion

The aim of this study was to examine population-average differences in change from baseline (T1) to post-intervention (T2) between the experimental and control conditions in a cluster-randomized trial evaluating the school-based gambling prevention program *What’s at stake?* (WAS?).

The results showed significant short-term differential changes between conditions in several outcomes, consistent with an immediate preventive effect at the population level.

Gambling intention showed a significant short-term differential reduction in the experimental group. This finding is relevant, as intention constitutes a direct predictor of gambling behaviour according to the Theory of Planned Behaviour (Ajzen, 1991), suggesting a potential preventive effect that may require booster sessions to achieve long-term stability.

Subgroup analyses by gender and prior gambling experience should be considered exploratory and interpreted with caution, as they were based on reduced sample sizes and were not powered to detect differential intervention effects. Accordingly, these findings cannot be regarded as confirmatory evidence of subgroup-specific effectiveness. Regarding gender, similar patterns have been reported by Donati et al. (2014) and Neighbors et al. (2015), who showed that universal interventions can modify gambling intentions and related beliefs across genders, albeit with some nuances. Regarding prior gambling experience, the pattern of results indicated non-significant but more favourable changes among adolescents who had not yet initiated gambling, which is consistent with the preventive focus of universal interventions.

Descriptively, at the population level, the proportion of students classified as at-risk or problem gamblers decreased more markedly in the experimental condition immediately after the intervention. Given that the SOGS-RA assesses gambling behaviour over the previous 12 months, this marked short-term reduction observed at post-intervention should not be interpreted as an actual behavioural change. Rather, it likely reflects a shift in self-perception, problem awareness, and cognitive appraisal following the intervention, a pattern commonly reported in brief prevention programs (Monreal-Bartolomé et al., 2023). Although this pattern is descriptive rather than a sustained population-average effect, it is nonetheless relevant because it suggests that the program was able to produce short-term shifts in risk classification, which is a key proximal objective in universal prevention and a necessary preliminary step for longer-term change. This immediate shift is comparable to the findings of Chóliz et al. (2021) in their evaluation of a prevention program in Spanish adolescents, although their study did not include a control group or follow-up measures.

Regarding gambling motives, short-term model showed significant differential reductions in all motives, reflecting a containment effect in the experimental group. The differential changes observed between conditions are consistent with a population-level preventive effect, particularly in reducing the belief that gambling is a viable way of obtaining economic profit. This finding is consistent with Donati et al. (2014), who reported that preventive interventions are especially effective in modifying erroneous beliefs about the probability of winning. The reduction in enhancement/pleasure motivation is also aligned with the results of previous studies (Keen et al., 2017; Neighbors et al., 2015).

Regarding risk perception, a significant Condition × Time interaction emerged in the short term (T_1_–T_2_). The control group showed a marked increase, whereas the experimental group remained relatively stable, with a slight decrease in this reverse-scored indicator. This finding aligns with previous studies, which typically reported immediate improvements in knowledge and risk perception (Forsström et al., 2021). However, our pattern does not reflect a clear intervention-related improvement, as the significant interaction was driven by an increase in the control group, and contrast with studies reporting immediate gains in risk perception, highlighting the resistance of this construct to short-term change during adolescence. Resistance to change in risk perception must be interpreted in the context of developmental factors, such as the maturational gap between the limbic system and the prefrontal cortex (Casey et al., 2008; Steinberg, 2010), dopaminergic hypersensitivity (Spear, 2000), and characteristic adolescent cognitive patterns, such as the personal invulnerability bias (Elkind, 1967). These developmental dynamics make adolescence a stage of heightened sensitivity to sensation seeking, which generally reduces perceived risk.

Regarding the control group, the increase in risk perception may reflect an assessment reactivity effect, whereby repeated measurement raises awareness and elicits more reflective responses even without intervention. This pattern is consistent with previous prevention studies and points to increased problem awareness rather than a true intervention effect. This interpretation is consistent with the small effect magnitudes observed and with the short interval between assessments.

Regarding normative perception, its inclusion is justified by evidence showing that when a behaviour is perceived as frequent or common, the likelihood of engaging in it increases significantly (Cunningham et al., 2012; Larimer & Neighbors, 2003). This misperception is particularly elevated among medium-frequency gamblers or those initiating gambling (Raisamo & Lintonen, 2012), and has been associated with lower risk perception and higher gambling intention (Donati et al., 2014; Shead et al., 2010).

Normative perception also showed short term effects. This suggests that the intervention was effective in decreasing the perceived normality associated with gambling behaviour, reducing the initial higher normative perception observed in the experimental group. This result aligns with previous studies in which correcting normative misperceptions reduced gambling intention. These findings are consistent with Ladouceur et al. (2013), who demonstrated that correcting misperceptions of peer gambling prevalence can reduce gambling intention.

This study is not without limitations. First, the magnitudes observed were small to moderate, which is consistent with prior literature on gambling prevention programs (Forsström et al., 2021; Monreal-Bartolomé et al., 2023). Although the design included control group, a limitation is the absence of an adequately powered follow-up assessment in the revised analyses, which prevents conclusions about medium- or long-term maintenance of effects. Therefore, conclusions are limited to short-term population-level effects. Furthermore, a key methodological limitation of this study is the high attrition rate observed between measurement points. Although the loss of participants in the experimental group between baseline and post-intervention was 14%, which is generally considered acceptable for school-based prevention trials, it may still have reduced statistical power for some subgroup analyses.

Future research should incorporate strategies to reduce attrition, include fidelity metrics and cluster-level process indicators to further minimize bias and strengthen generalizability. Although we used Spanish adolescent-validated versions of the GMQ (Grande-Gosende et al., 2019), the financial subscale added from the GMQ-F (Dechant, 2013) has not yet been formally validated in Spanish adolescents. This lack of specific validation for the financial items represents a methodological limitation of our study. In addition, because the SOGS-RA assesses gambling behaviour over the previous 12 months, the short-term reduction observed at post-intervention cannot be interpreted as a direct behavioural effect.

Regarding implementation, material fidelity was monitored informally, without systematic session-level records. Despite facilitators receiving structured training and weekly supervision, this lack of formal monitoring is acknowledged as a limitation. It should also be noted that the study did not incorporate family or contextual factors, which may play an important role in adolescent gambling behaviour and, therefore, in the effectiveness of the program. Nevertheless, the favourable direction of the changes for gambling intention suggests a potential preventive effect that warrants reinforcement through booster sessions (Monreal-Bartolomé et al., 2023).

### Conclusion

Overall, these results compare favourably with those reported in many previous studies. For example, the review by Keen et al. (2017) found that only five of fifteen programs evaluated achieved significant changes in gambling behaviour. In conclusion, the *What’s at stake?* program produced significant short-term population-level effects on key cognitive and motivational risk factors. Effect magnitudes were modest but consistent with the previous literature. Short-term reductions in gambling motivations, risk and normative perceptions, and gambling intention highlight the preventive potential of the intervention, thereby helping to address a major gap in the evidence base on school-based gambling prevention in Hispanic adolescent populations.
